# Use and knowledge of contraceptive methods by patients in two substance use disorders treatment centers in Paris

**DOI:** 10.1186/s12954-017-0181-y

**Published:** 2017-08-04

**Authors:** Virgile Clergue-Duval, Suzanne Robin, Maeva Fortias, Gaël Dupuy, Béatrice Badin-de-Montjoye, Florence Vorspan

**Affiliations:** 1 Faculté de Médecine, Université Paris Diderot, Sorbonne Paris Cité, Paris, France; 2 Master Genre, Politique et Sexualité, Ecole des Hautes Etudes en Science Sociale, Paris, France; 3 CSAPA Espace Murger, Service de Médecine Addictologique, Hôpital Fernand Widal, APHP, Paris, France; 4 CSAPA Centre Cassini, Service de Psychiatrie, Hôpital Cochin, APHP, Paris, France

**Keywords:** Contraception, Addiction, Contraceptive prevalence surveys, Substance use disorders treatment centers

## Abstract

**Background:**

Studies on contraceptive use by patients with substance use disorders (SUD) show a concerning low use of contraception. Mainly conducted in USA, they could be irrelevant to patients attending European SUD treatment centers, especially since these studies mostly investigate women suffering from social exclusion, severe material deprivation andopiates use with frequent high-risk drug use and sexual behaviors including sex trade, frequently not currently attending treatment centers. The purpose of this study is to describe contraceptive use by patients, both male and female, since contraception can not only be considered as a female problem, with severe SUD in two free clinics in Paris, France.

**Methods:**

An anonymous self-report questionnaire was distributed to literate patients followed in two generalist substance use disorders treatment centers in hospitals of Paris, France: *Espace Murger* and *Centre Cassini*, during 5 weeks between February and March 2016.

**Results:**

Out of the 78 respondents (with an age mean 40.7 years, in which women are represented as 48.1%, and 29.7% of them have children), 53 have had at least one sexual partner in the last 6 months. Contraception was “always” used by 55.3% of sexually active patients, “sometimes” by 19.1%, and “not” used by 25.5%. Male condoms were the main contraceptive method. The use of intrauterine devices was low, contrarily to what is observed in the French general population. However, the knowledge of contraceptive methods was common.

**Conclusions:**

In this population, with a high prevalence of at risk sexual behavior, the use of contraceptive methods is lower than in French general population. During standard care for SUD, contraception and desire to be a parent should be discussed and patients empowered to make their own choices. Lack of knowledge does not seem to be a hindrance to the use of contraception, but other sociological, psychological, or medical factors may limit contraceptive access and long-term use, especially for the long-acting reversible contraception methods. It is necessary to further develop this reflection by discussing the individual contraceptive choices with the patients themselves to clarify the nature of these constraints and maybe provide several contraceptive methods within the SUD care settings.

## Background

The medical contraception was a major breakthrough in fertility control. In France, the availability of modern contraceptive methods is theoretically guaranteed. All general practitioners are expected to counsel and to prescribe them. They are provided by pharmacies on medical prescription and partially supported by the public health insurance (hormonal pills, intrauterine devices, implant). Furthermore, access to general practitioners is free for patients with low income and non-citizens (through Couverture Maladie universelle and Aide médicale d’état programs). Despite that, precarious populations have weaker effective access to contraception [1]. Among them, French public health institutions assert the need for interventions on contraception and gynecology for patient with substance use disorders (SUD) [2]. International studies show they use contraceptives less often, choose male condoms most often, and have a lack of knowledge of contraceptive methods available [3–7]. These studies mainly focus on women suffering from social exclusion and material deprivation, mainly users of opiates with high-risk drug use and sexual behaviors including sex trade and those not currently enrolled with treatment centers [3]. However, these studies are not easily applicable to all patients of substance use disorders treatment centers nor assessing their needs of family planning services. They were conducted mainly outside of Europe (USA or Australia) [3]. Plus, contraceptive habits vary by country. In France, the latest available study (2006) focused exclusively on women with intravenous use and infection by the human immunodeficiency virus, which is not the majority of the patient population followed in care centers [8]. In addition, preventing contraceptive failures also involves informing and empowering men and so they should be included in studies [9].

Our main objective was to describe the use of contraceptive methods of patients followed in substance use disorders treatment centers in Paris, France. Our secondary objectives were to describe patients’ knowledge of contraceptive methods and to estimate the rate of abortion.

## Methods

This study was observational and cross-sectional. The study population included all French-speaking, literate patients (both women and men) followed in two generalist substance use disorders treatment centers in university hospitals of Paris, France: *Espace Murger* and *Centre Cassini*. Both situated within university hospitals (Fernand Widal and Cochin), those centers are funded to provide free medical, psychological and social care for adult patients with SUD, including inpatient withdrawal programs, including also on site as well as take home heroin maintenance treatment delivery (buprenorphine or methadone) even in patients who can be noncitizens or without medical insurance, or not enrolled in Couverture Maladie Universelle and Aide médicale d’état programs. Those centers can also provide medication to treat medical conditions. Those centers have a legal obligation to guaranty anonymity if a patient wishes it. Although abstinence- or maintenance-oriented, those centers are familiar with harm reduction approaches toward patients with SUD and can provide needles, syringes, and crack smoking devices, as well as condoms.

To explore patients’ use and knowledge about contraception, we designed a two-sided page anonymous self-report questionnaire based on a literature review and formatted to entail minimum disturbance in the running of the services according to the staff of the centers. We used the formulation and categorization of the French National Institute for Demographic Studies (INED) and Santé Publique France, the national research and prevention institute. The questionnaire explored different contraceptive methods including condoms, the pill, intrauterine devices (IUD), sterilizations, implants, injections, the patch, the ring, spermicide, diaphragms, caps, periodic abstinence, withdrawal, and sex without vaginal penetration. Medical abortion, unplanned pregnancy, and emergency contraception were also investigated.

The questionnaire was distributed by the reception staff to patients, both women and men, coming for medical visit or heroin maintenance treatment delivery, during 5 weeks between February and March 2016. An envelope was joined with it to ensure anonymity. Participation was voluntary and a refusal had no repercussions on patient care. The data was analyzed with the R program.

## Results

Seventy-eight patients completed the survey, aged from 18 to 54 (mean 40.7 years, see Table 1 for patient’s characteristics). During these 5 weeks, approximately 600 medical consultations took place in *Espace Murger* and 400 at the *Centre Cassini* so the participation can be estimated between 5 and 10% of the total visitors.

**Table 1:** Characteristics of patients who responded (number of patients)

Center	*n* = 78
Centre Cassini	24
Espace Murger	54
Gender	*n* = 77
Female	37
Male	40
General practitioner	*n* = 74
No	19
Yes	55
Doctor to discuss and prescribe contraception	*n* = 75
No	29
Yes	46
Personal situation (<6 months)	*n* = 73
Casual partner(s)	14
No partner	21
Regular partner	31
Regular and casual partners	4
Did not respond	3
Children	*n* = 74
No	52
Yes	22
First product or circumstances that motivated the request for treatment	*n* = 78
Alcohol	24
Behavioral addiction	4
Cannabis	15
Cocaine	14
Crack	6
Heroin/opiate	34
Prescribed drugs	16
Tobacco	8

Among the 53 patients with at least one partner in the last 6 months and unsterilized, 26 declared “always” using contraception (55.3%), nine “sometimes” (19.1%), and 12 “never” (25.5%). The contraceptive methods used were the male condom alone for 21 patients (39.6%), the hormonal pill for seven (13.2%), IUD for three (5.7%), an implant for two (3.8%), the ring for one. Two patients (3.8%) used the male condom combined with another method including a man using withdrawal as alternative according to the situations.

Among respondents, 89.0% (65) knew of emergency contraception, 37.5% (27) have used it, 35.6% (26) were ever confronted with an unplanned pregnancy, and 41.1% (30) with a medical abortion (themselves or their partner) during their life. Nine (12.3%) reported having had a medical abortion in their lifetime yet not having faced an unplanned pregnancy. The main contraceptive methods previously used by the patients were the male condom (89.2% of 74 respondents) and the pill (63.5%). Among other methods, withdrawal was used by 35.1% (26 patients), IUDs by 18.9% (14), sex without vaginal penetration by 16.2% (12), implant by 10.8% (8), and periodic abstinence (Billing, Ogino…) by 10.8% (8).

On average, patients knew of 9.7 contraceptive methods of the 15 proposed (standard deviation = 4.0). The results are displayed in Table 2.

**Table 2:** The knowledge of contraceptive methods

Contraceptive method	Yes, sufficiently	Yes, insufficiently (%)	No (%)
Cap	5.8% (*N* = 4)	14.5	79.7
Diaphragm	36.1% (*N* = 26)	26.4	37.5
Female condom	42.5% (*N* = 31)	35.6	21.9
Female sterilization	38.9% (*N* = 28)	36.1	25.0
Hormonal Pills	76.7% (*N* = 56)	16.4	6.8
Implant	34.7% (*N* = 25)	20.8	44.5
Injection	13.7% (*N* = 10)	21.9	64.4
Intra-uterin device (IUD)	50.0% (*N* = 34)	29.4	20.6
Male condom	91.8% (*N* = 67)	6.8	1.4
Male sterilization	36.1% (*N* = 26)	37.5	26.4
Patch	27.4% (*N* = 20)	21.9	50.7
Periodic abstinence	29.6% (*N* = 21)	31.0	39.4
Ring	30.1% (*N* = 22)	31.5	38.4
Spermicide	29.2% (*N* = 21)	27.8	43.0
Withdrawal	66.2% (*N* = 47)	19.7	14.1

Percentages are given for responders only

*N* number of patients who responded “Yes, sufficiently”

## Discussion

Our response rate was lower than expected, showing the difficulty of approaching this issue. Participants corresponded to the target population in terms of age and substances used, but unsurprisingly, women were more likely to respond. Despite these biases, we have shown relevant elements for practitioners. Firstly, contrary to what we expected, we found similar observations to previous studies on the most precarious patients, those at the margins of the care system. The patients in our study used contraception less than the general population in France (74.5 vs 96.9%) [10, 11]. On the other hand, this rate is similar to the high range of Terplan’s meta-analysis (25–77%) (3). This may reflect the combined severity of the SUD as well as high access to medical and social care for patients attending this type of free clinics. Secondly, we noticed a deviation from the typical path of contraception observed in the general population. Usually, younger women tend to use the pill and the trend changes after pregnancies or passed the age of 35, when IUDs become more popular [10]. Our respondents do not follow this pattern with most of them using male condoms despite a mean age of 41 years. The condom is the method used to prevent sexually transmitted infections (STI). It is not surprising that its use may be more widespread than in general population and demonstrates the good acceptance of this method in our population. However, it is also widely used by regular and stable partners in our sample. We make the hypothesis that in our population, condom is a way of preventing STI even with a regular partner known as HIV or HCV positive or to avoid to have to deal with the potential risky behavior of drug use. Condom also allows real-time control over contraception by patients. But condom alone is not the best effective contraceptive method in practice compared to the long-acting reversible contraception (LARC) as IUD or the implant [12]. Thirdly, in our sample, we describe a knowledge of the various contraceptive methods similar to that observed in the general population in France [13]. If the insufficient knowledge is the first reason raised by studies [3, 14], there are others that may influence the contraceptive choices as the practice availability or the absence of personal support and advice [14, 15]. The cost is possibly less an influence factor than in other countries [16] since in France, the majority of contraceptive methods are defrayed for all patients by public medical insurance, except the condom, which is freely available in our centers. In France, the main obstacle to the IUD use in general population is the lack of trained professionals to install them [17]. There may be some reluctance regarding LARC and a mistrust of the medical supervision they induce [14, 15, 18]. The use of contraception appears to be a patient’s deliberate choice, and it is necessary to further investigate the reasons for a given choice through individual interviews [15]. Lastly, it is particularly striking to observe that while some patients declare “planning” pregnancies, they practice abortion de facto. This questions what influences them upon deciding whether to abort. As with all chronic diseases, we must include addiction care in the life course of patients [19, 20]. It is difficult to describe the contraceptive choices without attempting to understand the desire to be a parent, sometimes latent, and support couples in their choice of planning parenthood [21, 22].

## Conclusions

In both centers, the use of contraceptive methods is not as low as previously observed in other populations with SUD, but it appears lower than the general population. We observed a common knowledge of contraceptive methods and a dominant use of condom with little use of IUDs by patients of 41 years at mean.

During standard care for SUD, contraception and desire to be a parent should be discussed and patients empowered to make their own choices. Lack of knowledge does not seem to be a hindrance to the use of contraception, but other sociological, psychological, or medical factors may limit contraceptive access and long-term use in this specific population, especially for the long-acting reversible contraception methods. It is necessary to further develop this reflection by discussing the individual contraceptive choices with the patients themselves to clarify the nature of these constraints and maybe provide several contraceptive methods within the SUD care settings.

## Abbreviations

IUD: Intrauterine devices
LARC: Long-acting reversible contraception
STI: Sexually transmitted infections
SUD: Substance use disorders
